# TFII-I/Gtf2i and Erythro-Megakaryopoiesis

**DOI:** 10.3389/fphys.2020.590180

**Published:** 2020-09-25

**Authors:** Aishwarya Gurumurthy, Qiong Wu, Rukiye Nar, Kimberly Paulsen, Alexis Trumbull, Ryan C. Fishman, Marjorie Brand, John Strouboulis, Zhijian Qian, Jörg Bungert

**Affiliations:** ^1^Department of Biochemistry and Molecular Biology, College of Medicine, UF Health Cancer Center, Genetics Institute, Powell Gene Therapy Center, University of Florida, Gainesville, FL, United States; ^2^Division of Medicine and Department of Biochemistry and Molecular Biology, UF Health Cancer Center, University of Florida, Gainesville, FL, United States; ^3^Sprott Center for Stem Cell Research, Ottawa Hospital Research Institute, Ottawa, ON, Canada; ^4^Comprehensive Cancer Center, School of Cancer and Pharmaceutical Sciences, Faculty of Life Sciences and Medicine, King’s College London, London, United Kingdom

**Keywords:** transcription factor, TFII-I, Gtf2i, erythropoiesis, megakaryopoiesis, globin

## Abstract

TFII-I is a ubiquitously expressed transcription factor that positively or negatively regulates gene expression. TFII-I has been implicated in neuronal and immunologic diseases as well as in thymic epithelial cancer. Williams–Beuren Syndrome (WBS) is caused by a large hemizygous deletion on chromosome 7q11.23 which encompasses 26–28 genes, including *GTF2I*, the human gene encoding TFII-I. A subset of WBS patients has recently been shown to present with macrocytosis, a mild anemia characterized by enlarged erythrocytes. We conditionally deleted the TFII-I/*Gtf2i* gene in adult mice by tamoxifen induced Cre-recombination. Bone marrow cells revealed defects in erythro-megakaryopoiesis and an increase in expression of the adult β-globin gene. The data show that TFII-I acts as a repressor of β–globin gene transcription and that it is implicated in the differentiation of erythro-megakaryocytic cells.

## Introduction

Transcription factor TFII-I (gene symbols *GTF2I* in human and *Gtf2i* in mice) is a ubiquitously expressed multifunctional protein and contains a basic DNA-binding domain, a nuclear localization sequence, and multiple protein–protein interaction domains, including a leucine zipper and 6 R-repeats (Roy, 2001, 2012). The R-repeats resemble helix-loop-helix (HLH) domains typically found in E-box binding proteins and TFII-I has been demonstrated to interact with HLH proteins including c-Myc and USF (Roy et al., 1991, 1993; Doi-Katayama et al., 2007). TFII-I haploinsufficiency is associated with Williams–Beuren Syndrome, which is characterized by cranio-facial and neurological defects (Tassabehji et al., 2005; Pober, 2010). Ablation of TFII-I function in mice caused early embryonic lethality due to defects in vasculogenesis and angiogenesis (Enkhmandakh et al., 2009). TFII-I positively or negatively regulates transcription of specific genes through either initiator elements or other *cis*-regulatory DNA-binding sequences (Roy, 2001, 2012; Anantharaman et al., 2011). Furthermore, TFII-I interacts with the chromatin insulator protein CTCF and regulates a number of genes in response to metabolic stress (Peña-Hernández et al., 2015).

Previous genomic and proteomic analysis of TFII-I revealed that it interacts downstream of Pol II peaks at several stress response genes in human erythroleukemia K562 cells (Fan et al., 2014). Among stress induced genes bound by TFII-I in erythroid cells are the *ATF3* gene and the gene encoding the heme-regulated inhibitor (HRI), an eIF2 α kinase that inhibits translation in response to heme depletion (Chen, 2014). Both ATF3 and HRI have been implicated in the regulation of globin gene expression (Suragani et al., 2012; Hahn and Lowrey, 2013; Grevet et al., 2018). In addition to regulating genes encoding cell cycle modulators or genes responsive to stress, we found that TFII-I interacts with erythroid-specific gene promoters, including that of β-globin and GATA1 (Leach et al., 2003; Fan et al., 2014).

In this study we examined the role of TFII-I during hematopoiesis in mice. We obtained mice that allow conditional (Cre-mediated) ablation of TFII-I gene function (Enkhmandakh et al., 2016) and analyzed bone marrow hematopoiesis after induction of Cre-recombination. The data show that TFII-I deficiency causes an increase in expression of the adult β-globin gene and a mild defect in erythropoiesis. Importantly, we observed an approximately twofold decrease in the formation of megakaryocytes in mice deficient for TFII-I.

## Materials and Methods

### Mice

We purchased two mouse strains from the Jackson Laboratory. One strain contains two *loxP* sites surrounding exon 3 of the Gtf2i gene (B6;129S-Gtf2i^TM1^.^1Bdash^/J, referred to as **Gtf2i-floxed**) and another which expresses a Cre-ER fusion protein that translocates to the nucleus in response to tamoxifen [B6.Cg-Tg(CAG-cre/Esr1^∗^)5Amc/J], referred to as **CAGGCre-ER^TM^**. The mice were mated to generate heterozygous KO/Gtf2i-floxed mice that are either positive or negative for the Cre gene. The following DNA primers where used for genotyping: GTF2I US: 5′TGTTAGGGCAGGTGATGA, DS: 5′AGCCACTCCAACAGTTACCG; CAG-Cre US: 5′GCTAACC ATGTTCATGCCTTC, DS: 5′AGGCAAATTTTGGTGTACGG. These mice were subjected to intraperitoneal tamoxifen injection (100 μl of a 20 mg/ml tamoxifen solution in corn oil for 10 days).

### Extraction of Bone Marrow Cells

The bone marrow was isolated using a previous published protocol (Liu and Quan, 2015). Briefly, the hind leg femurs were cut at both ends and, using a 23-gage needle and a 10c syringe filled with ice-cold HBSS, the bone marrow was flushed into a 50 ml Falcon conical tube. The cells were centrifuged at 1500 rpm for 7 min at 4°C. The cell pellet was resuspended with 1 ml RBC lysis buffer, incubated at room temperature for 5 min, and the lysis buffer was neutralized by adding 5 ml FBS. The cells were centrifuged at 100 rpm for 7 min at 4°C, and the cell pellet was used for further downstream analysis.

### RT-qPCR

RNA was extracted using the RNeasy kit (Qiagen, Hilden, Germany), and reverse transcribed into cDNA using the IScript cDNA synthesis kit (Bio-Rad). The cDNA was subjected to qPCR in a 10-μl reaction mix with SYBR green. Gene expression was analyzed by the normalization of expression to that of GAPDH using the ΔCT method. The following DNA primers were used: βmajor Forward: CACATTTGCTTCTGACATA, βmajor Reverse: GCAGAGGCAGAGGATAGGTC; α-globin Forward: CCTGGGGGAAGATTGGTG, α-globin Reverse: GCCGTGGCTTACATCAAAGT; GAPDH Forward: TGGTGA AGGTCGGTGTGAAC, GAPDH Reverse: CCATGTAG TTGAGGTCAATGAAGG; TFII-I Forward: CCAGAGCTGC TCACTCACAG, TFII-I Reverse: GATCTCTTCCACTTGCT TTCG.

### Flow Cytometry

Bone marrow cells were subjected to flow cytometry analysis using antibodies against CD71 and Ter119 for monitoring erythroid differentiation, and CD41 for monitoring differentiation of megakaryocytes. Flow cytometry was performed without cell lysis. These experiments were performed according to published procedures (Wang et al., 2010; Zhang et al., 2018).

## Results

**FIGURE 1:**
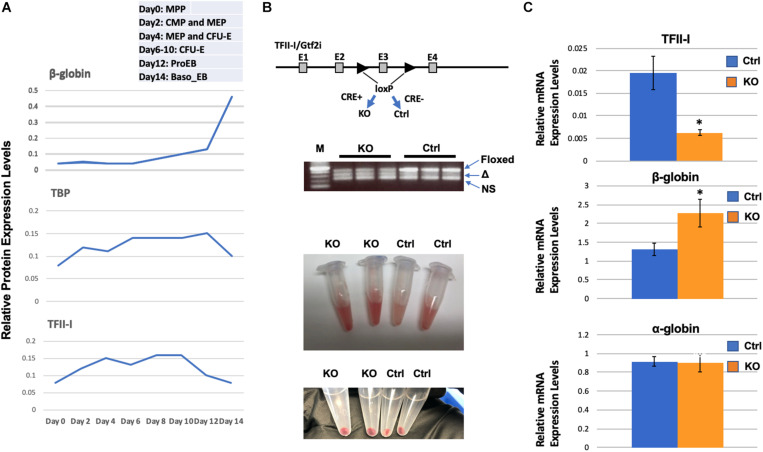
Enhanced β-globin gene expression in TFII-I/Gtf2i deficient mice. **(A)** Relative protein expression levels for TFII-I, β-globin, and TBP during differentiation of erythroid cells. Data were extracted from a proteomic study by Gillespie et al. (2020). MPP, multiple progenitor population; CMP, common myeloid progenitors; MEP, myeloid erythroid progenitors; CFU-E, colony forming unite-erythroid; ProEB, proerythroblast; Baso EB, basophilic erythroblast. **(B)** *Gtf2i* floxed mice were mated with mice expressing Cre-recombinase in a tamoxifen inducible manner. PCR bands from three KO and three control mice represent the floxed Gtf2i gene (Floxed, 410 bp), the exon 3 deleted TFII-I gene (Δ, 328 bp), and a non-specific band (NS); lane M shows 500, 400, 300, 200, and 100 bp DNA fragments from top to bottom. Shown below are bone marrow cells and cell pellets from knock-out (KO) or control (Ctrl) mice. **(C)** RT-qPCR analysis of TFII-I, β-globin, and α-globin expression in bone marrow cells from KO and control mice (C1). Error bars are derived from two experimental replicates and three technical (qPCR) replicates (*p < 0.05).

We have previously shown that TFII-I interacts with HDAC3 and functions as a repressor of adult β-globin gene expression in erythroleukemia cell lines (Crusselle-Davis et al., 2006). Gillespie et al. (2020) recently performed a proteomic study in primary differentiating erythroid cells. The relative expression data for TFII-I demonstrate that it is up-regulated during differentiation of erythroid cells and declines in expression concomitant with the up-regulation of adult β-globin gene expression (Figure 1A). As a comparison, expression of the TATA-binding protein (TBP) also declines in expression but at a later stage. Analysis of proteomic data from Gautier et al. (2020) revealed similar results; TFII-I expression declines during differentiation of erythroid cells at the same time at which expression of β-globin increases.

We generated transgenic mice that have exon 3 of the Gtf2i gene flanked with *loxP* sites and that express the Cre-recombinase in a tamoxifen inducible manner (Figure 1B). In these mice, Cre is fused to the estrogen receptor (ER) and tamoxifen induces nuclear localization. We injected mice positive for Cre and the floxed TFII-I gene locus or as controls, mice that only have the floxed TFII-I gene locus but do not express the inducible Cre. We consistently observed that the bone marrow cells from TFII-I KO mice had a more intense red color compared to cells from control mice (Figure 1B). We subjected cells from bone marrow to RT-qPCR analysis. We found that TFII-I expression was reduced fourfold in the Cre-expressing mice compared to the Cre-negative mice (Figure 1C). Furthermore, TFII-I deficient bone marrow cells exhibited a twofold increase in expression of the adult β-globin gene. However, there was no change in expression of the α-globin genes.

**FIGURE 2:**
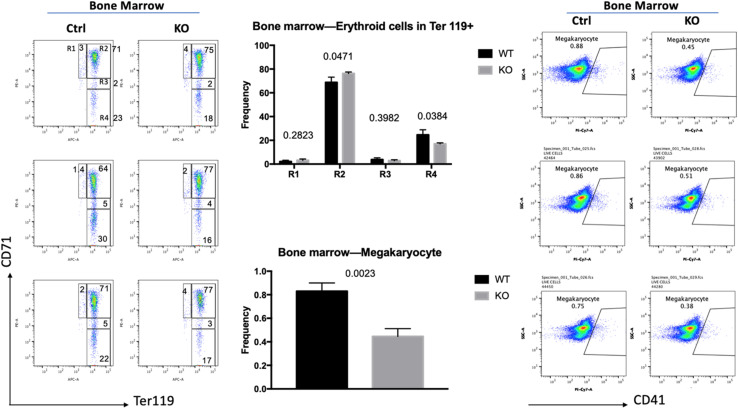
Flow cytometry analysis of TFII-I KO and control bone marrow cells. BM cells isolated from three KO and three control mice (Ctrl) were subjected to flow cytometry analysis using CD71 and Ter119 antibodies (left) or CD41 antibodies (right). The numbers on top of the bars in the middle represent the *p*-values (error bars derived from three KO and three control mice, *n* = 3; *p*-values are shown on top of the graphs in the middle).

To investigate if the increase in globin gene expression was due to changes in the differentiation profile of the erythroid cells, we examined bone marrow cells by flow cytometry (Figure 2). Previous studies established that CD71, the transferrin receptor, and Ter119 antigens are useful markers for the discrimination of different erythroid maturation stages (Liu et al., 2013). The R1 population (CD71 high, Ter119 low) represents immature erythroid cells, while R2 (CD71 high, Ter119 high), R3 (CD71 intermediate, Ter119 high), and R4 (CD71 low, Ter119 high) represent increasing stages of erythroid maturation. We injected six mice with tamoxifen (three Cre-positive and three Cre-negative mice) for 10 days. Five days after the last injection the mice were sacrificed, and cells were isolated from bone marrow. TFII-I deficient mice revealed a mild but statistically significant change in the differentiation profile of erythroid cells. The R1 population was increased by 50%, the R2 population was increased by 11%, the R3 population was decreased by 34%, and the R4 population was decreased by 35% in TFII-I KO versus control mice. The changes in the R2 and R4 populations were statistically significant with *p*-values of 0.047 and 0.038, respectively. Moreover, we did observe an about twofold decrease in the number of CD41, integrin alpha chain 2b, positive mature megakaryocytes (47% reduction, *p*-value of 0.0023). These data show that TFII-I deficient mice exhibit a defect in erythro-megakaryocytic cell differentiation.

## Discussion

The data presented here demonstrate that TFII-I deficiency results in an increase in adult β-globin gene expression. This observation is consistent with previous data from our laboratory showing that TFII-I interacts with the adult β-globin gene in K562 cells, in which the gene is not expressed, and that expression of a dominant negative TFII-I in K562 cells enhanced expression of β-globin, while expression of TFII-I in differentiated MEL cells reduced adult β-globin gene expression (Crusselle-Davis et al., 2006). TFII-I expression changes during differentiation of erythroid cells. Data from the Brand laboratory show that TFII-I expression increases during the early stages of erythroid differentiation and declines at later stages (Gillespie et al., 2020). The decline in TFII-I expression parallels an increase in expression of β-globin at the later stages of differentiation. Interestingly, we did not observe an increase in α-globin gene expression in TFII-I deficient bone marrow cells. Although expression of the globin genes is coordinated, the α- and β-globin loci are in different chromosomal environments (Higgs et al., 2012). While the α-globin locus is in a constitutively open chromatin environment, accessibility of the β-globin genes is restricted to erythroid cells. Perhaps TFII-I prevents premature β-globin expression during the process of chromatin opening in differentiating erythroid cells.

We found that TFII-I deficiency also impaired differentiation of erythroid cells. We detected a statistically significant increase in immature erythroid cells and a decrease in the number of mature erythroid cells. It should be noted that ablation of TFII-I expression was not complete. Complete TFII-I deficiency during erythroid differentiation would be predicted to have a stronger effect on the differentiation of erythroid cells. Perhaps related to our results, a recent study demonstrated that at least a subset of Williams Syndrome patients exhibit mild macrocytic anemia, characterized by enlargement of red blood cells (Yu et al., 2020). Megaloblastic macrocytosis is characterized by slow DNA synthesis, which is interesting with respect to previous data implicating TFII-I in the regulation of genes encoding cell cycle modulators (Roy, 2001, 2012).

We also observed a decrease in the number of megakaryocytes, which produce platelets. Erythroid and megakaryocytic cells have common progenitors (Pimkin et al., 2014), suggesting that TFII-I acts on erythro-megakaryocytic progenitors. The same progenitor cells are also involved in generating endothelial cells (Plein et al., 2018). It is thus interesting to note that complete TFII-I deficiency causes early embryonic lethality associated with defects in vascularization and angiogenesis (Enkhmandakh et al., 2009).

In summary, our data implicate TFII-I in the regulation of adult β-globin expression and in the specification of erythro-megakaryocytes.

## Data Availability Statement

The original contributions presented in the study are included in the article/supplementary material, further inquiries can be directed to the corresponding author.

## Ethics Statement

The animal study was reviewed and approved by University of Florida IACUC.

## Author Contributions

AG, JS, ZQ, RN, and JB designed the experiments. AG, QW, KP, AT, and RF performed the experiments. MB contributed data. AG and JB wrote the manuscript with impact from JS and ZQ. All authors contributed to the article and approved the submitted version.

## Conflict of Interest

The authors declare that the research was conducted in the absence of any commercial or financial relationships that could be construed as a potential conflict of interest.
